# Swiss University Students’ Attitudes toward Pharmacological Cognitive Enhancement

**DOI:** 10.1371/journal.pone.0144402

**Published:** 2015-12-10

**Authors:** Larissa J. Maier, Evangelia Liakoni, Jan Schildmann, Michael P. Schaub, Matthias E. Liechti

**Affiliations:** 1 Swiss Research Institute for Public Health and Addiction (ISGF), Associated Institute at the University of Zurich and WHO Collaborating Centre, Zurich, Switzerland; 2 Division of Clinical Pharmacology and Toxicology, Department of Clinical Research, University Hospital Basel, Basel, Switzerland; 3 Institut für Medizinische Ethik und Geschichte der Medizin, Ruhr-Universität Bochum, Bochum, Deutschland; Penn State College of Medicine, UNITED STATES

## Abstract

Pharmacological cognitive enhancement (PCE) refers to the nonmedical use of prescription or recreational drugs to enhance cognitive performance. Several concerns about PCE have been raised in the public. The aim of the present study was to investigate students’ attitudes toward PCE. Students at three Swiss universities were invited by e-mail to participate in a web-based survey. Of the 29,282 students who were contacted, 3,056 participated. Of these students, 22% indicated that they had used prescription drugs (12%) or recreational substances including alcohol (14%) at least once for PCE. The use of prescription drugs or recreational substances including alcohol prior to the last exam was reported by 16%. Users of pharmacological cognitive enhancers were more likely to consider PCE fair (24%) compared with nonusers (11%). Only a minority of the participants agreed with the nonmedical use of prescription drugs by fellow students when assuming weak (7%) or hypothetically strong efficacy and availability to everyone (14%). Two-thirds (68%) considered performance that is obtained with PCE less worthy of recognition. Additionally, 80% disagreed that PCE is acceptable in a competitive environment. More than half (64%) agreed that PCE in academia is similar to doping in sports. Nearly half (48%) claimed that unregulated access to pharmacological cognitive enhancers increases the pressure to engage in PCE and educational inequality (55%). In conclusion, Swiss students’ main concerns regarding PCE were related to coercion and fairness. As expected, these concerns were more prevalent among nonusers than among users of pharmacological cognitive enhancers. More balanced information on PCE should be shared with students, and future monitoring of PCE is recommended.

## Introduction

The nonmedical use of prescription drugs or recreational drugs to improve cognitive performance is referred to as pharmacological cognitive enhancement (PCE), which is often discussed in terms of ethical considerations and morality [1–4]. The public has raised strong concerns about the fairness and coercion of drug use for PCE [5].

Schelle et al. (2014) summarized evidence from 40 studies that contained information about public attitudes toward PCE. Aside from concerns regarding fairness and coercion, concerns were most often raised about the medical safety of substances that are used for PCE [5].

The prevalence of PCE among Swiss university students and their perceived pressure to use drugs for PCE were recently investigated by two web-based studies [6,7]. Both studies also included a small set of questions to examine students’ concerns about PCE in general, not differentiating between substances and different situations of use. Students worried about the medical safety of drugs that are used for PCE and reported perceiving peer pressure to use drugs for PCE [6,7]. Users of pharmacological cognitive enhancers were less concerned about side effects and potential disadvantages in those who refuse to use PCE compared with nonusers [7]. However, PCE among students might be considered cheating, resulting in inequality and injustice, potentially inauthentic results, or peer pressure to engage in PCE [5,7,8]. Information on attitudes toward PCE use was obtained with mostly small study samples, and only few empirical studies have assessed a wider range of students’ attitudes toward PCE in terms of the acceptability of the many different substances that are used for PCE [5,9]. Furthermore, only a few studies have compared PCE in academia with doping in sports [10–12]. Similar to a study among 1,026 German students, qualitative interviews among 19 Australian university students revealed that PCE was considered unfair, and monitoring and regulating PCE were rated as important [13,14]. In contrast, interviews with 18 healthy German students who used prescription drugs for PCE revealed that students see a moral difference between PCE with drugs and PCE with caffeine [9].

Most of the studies that have been performed to date were rather small and addressed only a few concerns regarding PCE in general and not specifically for different substances, as reported in a recent review [5]. Therefore, we addressed students’ attitudes toward various types of pharmacological cognitive enhancers among Swiss university students in a large, empirical, web-based survey, including quantitative ratings for each statement to allow statistical comparisons. We examined a series of specific statements regarding the acceptability of different drugs that are used for PCE and the implications of various situations of use. We also investigated attitudes concerning the authenticity of performance that is achieved with PCE, fairness, autonomy, and the need for regulation. We adjusted and included several questions that were used in previous interviews [10,13] and a quantitative study among German students [14] to compare our findings with previous international studies. We also investigated the frequency of the nonmedical use of prescription and recreational drugs [6] to statistically compare attitudes toward PCE between users and nonusers of pharmacological cognitive enhancers. Our primary hypothesis was that users of pharmacological cognitive enhancers would exhibit more accepting attitudes toward PCE compared with nonusers [5,7].

## Materials and Methods

### Recruitment of study participants

Students at the University of Zurich (UZH; total of 5,000 contacted), University of Basel (UniBas; total of 11,189 contacted), and Swiss Federal Institute of Technology Zurich (ETHZ; total of 13,093 contacted) were contacted via e-mail and invited to participate in an online survey on attitudes toward PCE. In the e-mail, potential participants were briefly informed that we were interested in students’ attitudes toward PCE, regardless of whether they were experienced with PCE or not. The participants were informed that participation in the study was voluntary, that responses would be anonymous and stored on a secure server, and that the study was authorized by the Ethics Committee of the Philosophical Faculty of the University of Zurich and Ethics Committee of the Cantons of Basel. All of the study participants provided informed consent when they left the study information webpage to start the survey. No incentives were provided for participation in the study. The participants were informed that completion of the questionnaire would take approximately 15 min.

### Measures (questionnaire)

The full study questionnaire is provided online (S1 Questionnaire). Question identifiers are included below in parentheses.

#### Definition of pharmacological cognitive enhancement

At the beginning of the questionnaire, PCE was defined as the use of prescription drugs (e.g., methylphenidate) or other psychoactive substances (e.g., alcohol, cannabis, and cocaine) to enhance cognitive performance (e.g., attention, concentration, vigilance, and reduction of nervousness) while studying, similar to our previous study [6]. Coffee and energy drinks were not considered for this narrow definition of PCE, but their rates of use and attitudes concerning their use were assessed for comparison.

#### Participant characteristics

The participants were first asked to indicate their (Q1) university, (Q2-4) study major, (Q5) number of semesters studied, (Q6) age, (Q7) sex, (Q8) whether they studied full-time or part-time, and (Q9) whether they worked (percentage of full-time employment).

#### Prevalence of pharmacological cognitive enhancement

Participants’ drug use that was aimed at improving study performance was assessed similarly to our previous study on PCE among Swiss university students [6]. Specifically, the participants were asked to indicate whether they had ever used one or more of the following (Q13) prescription drugs without a medical indication to improve cognitive performance while studying: methylphenidate, modafinil, antidepressants, anti-dementia drugs, sedatives/sleeping pills, and beta-blockers. They were also asked whether they had ever used any of the following (Q14) recreational drugs to improve cognitive performance while studying: alcohol, cannabis products, cocaine, illegal amphetamine, and methylenedioxymethamphetamine (MDMA). If the participants indicated that any substance was used explicitly for PCE, then they were subsequently asked (*i*) how often PCE occurred within the last 30 days prior to the last exam, (*ii*) whether their expectations regarding the efficacy of the substance that they used were met, and (*iii*) whether they would consider repeated use in a similar situation.

#### Attitudes toward pharmacological cognitive enhancement

The participants were asked to rate a series of statements that reflected different attitudes toward various types of PCE in various contexts of use. Several statements were adjusted from previous studies [13–15] and pilot-tested for comprehension and redundancy. The participants rated each statement on a Likert scale, indicating their agreement (1 = strongly disagree, 5 = strongly agree).

#### Substances used to improve cognitive performance while studying

First (Q15), the participants were asked to indicate their agreement with the use of one of the following substances: the prescription drug methylphenidate or modafinil with or without a prescription, illegal stimulants (e.g., amphetamine, cocaine, MDMA), coffee, caffeine tablets, and energy drinks. The participants were informed that they should assume that these substances generally have only weak effects on cognitive performance, in line with scientific data [16–18].

#### Case vignette: “Thomas”

Second (Q16), a “real-world” and prototypic vignette of PCE was presented that described a student, “Thomas,” who was studying for his final exam using non-prescribed methylphenidate. The example was taken from a previous qualitative interview study [15] and adjusted for the present study to allow for quantitative assessments. Thomas had concentration problems and complained to a colleague about feeling pressured and having problems learning the material for the exam. His friend recommended that Thomas should use methylphenidate to assist with studying and offered him some. Thomas used the methylphenidate and felt that it facilitated concentration over longer periods of time. He felt that he was more efficient at learning and less afraid of the exam. Thomas passed the exam with even better grades than previously. The participants were asked (*i*) whether they agreed with Thomas’ use of methylphenidate in this situation in general, (*ii*) whether it would have been acceptable to use methylphenidate with a doctor’s prescription but without a diagnosed disorder, and (*iii*) whether it would have been acceptable to use methylphenidate with a prescription and diagnosed attention deficit disorder.

#### Contexts of pharmacological cognitive enhancement, assuming hypothetically strong efficacy and legal availability

The participants then had to indicate their attitudes toward PCE with regard to different contexts of use, assuming that substances with strong cognitive-enhancing effects existed (which is currently not the case) that are legally available to everyone (Q17). Thus, the participants were asked whether they agreed with the use of an effective substance for cognitive enhancement (*i*) by any fellow students, (*ii*) by fellow students with bad grades, (*iii*) by fellow students who were diagnosed with a mental disorder (i.e., attention deficit), (*iv*) by fellow students with a mental disorder without a physician’s diagnosis, (*v*) by a surgeon to improve performance during long operations or night shifts, or (*vi*) by professors to enhance academic teaching/learning performance.

#### General attitudes toward pharmacological cognitive enhancement

The participants then rated a series of statements on general attitudes toward PCE. They were asked whether they agreed or disagreed (Q18) that PCE negatively alters the quality of an academic result (e.g., less authentic), (Q19) that substances that are used for PCE change emotions and social behavior of users, (Q20) that substances that are used for PCE change the personality of users, (Q21) that achievements with PCE are less worthy of recognition than similar achievements without substance use, (Q22) that someone who uses substances to study longer has planned his work poorly, (Q23) that substances that are used to improve studying hinder the acquisition of more sustainable planning and learning strategies, and (Q24) that mankind has always used substances to enhance performance (e.g., coffee, coca, betel, tobacco) and the use of modern substances, such as medications, is simply the most recent form of this phenomenon.

The participants then indicated (Q25) whether they considered PCE (25a) generally unproblematic and acceptable, (25b) unproblematic as long as the substances were safe, (25c) unproblematic as long as use is controlled and occasional, or (25d) unproblematic as long as no adverse effects caused harm to others (e.g., impaired driving skills).

#### Comparison with doping in sports

The participants were then asked (Q26) to compare PCE in academia with doping in sports using two partially contrasting statements. First, they had to indicate whether they considered PCE in academia as similar to doping in sports. Second, they were asked whether they agreed or disagreed with the statement that winners and losers exist in sports only, whereas in academia PCE that leads to success does not necessarily mean that others lose. Similarly, participants had to indicate (Q27) whether they consider PCE acceptable in a non-competitive environment where one’s own performance is not measured relative to someone else’s performance and in a competitive environment where one’s own performance is assessed relative to someone else’s performance.

#### Autonomy *vs*. regulation

Additionally (Q28), the participants shared their opinions about PCE and autonomy: (28a) individuals are autonomous, and everybody should be free to decide whether to use substances for cognitive enhancement or not, and (28b) they would not mind if other students took substances while studying. A set of statements then explored opinions about regulating PCE: (28c) the university should regulate substance use for the purpose of improving cognitive performance among students, (28d) unregulated access to substances that are used for cognitive enhancement might result in unequal chances with regard to education and professional life, (28e) unregulated access to substances that are used for cognitive enhancement increases pressure to use these substances (coercion), and (28f) access to medications and other substances is already sufficiently regulated; hence, specific regulations for PCE are unnecessary.

#### Duties of academic institutions

The participants were then asked (Q29) whether performance requirements at universities might increase because of PCE, (Q30) whether more investment in research that seeks to develop effective substances that increase cognitive performance is desirable, and (Q31) whether universities should collect data on the importance (prevalence/acceptance) of PCE among students to neutrally inform students about this topic and potential harms. Finally, the participants were asked (Q32) whether they would consider using substances for PCE that are safe and have few adverse effects, even if they would gain an advantage over others. The participants also had to indicate whether (Q33) they considered that PCE reflects media hype with no real relevance to society.

### Statistical analysis

Analyses were conducted using Statistica 12 software (StatSoft, Tulsa, OK, USA). Likert scales were used to measure attitudes toward PCE and are expressed as numeric scores: 1 = “strongly disagree,” 2 = “rather disagree,” 3 = “unsure,” 4 = “rather agree,” and 5 = “strongly agree.” To determine agreement (> 3) or disagreement (< 3) with a statement, the mean rating for each statement was tested against 3 using *t*-tests for single means. Differences in attitudes toward different types or contexts of PCE were assessed using nonparametric Friedman analyses of variance (ANOVAs) or Wilcoxon matched-pairs tests. Differences in attitudes between PCE users and nonusers were assessed using Kruskal-Wallis tests. The number (%) of participants who agreed or disagreed with a statement was determined for all statements with scores of 1 or 2 (indicating disagreement) and scores of 4 or 5 (indicating agreement). Multiple regression analysis was used to examine participant characteristics that predict agreement with the use of pharmacological cognitive enhancers in the case example (Q16). To account for multiple testing, the level of significance was set to *p* < 0.001 for all of the analyses.

## Results

### Participant characteristics

Response rates and participant characteristics are presented in S1 Table. A total of 3,056 students who completed at least the section on the use of pharmacological cognitive enhancers in the questionnaire were included in the study. The participants were 17–72 years old (mean, 23 years). The median number of completed semesters was six (range, 1–24). An equal number of male and female students participated in the study. Most of the participants from UniBas and UZH were female, and most of the participants from ETHZ were male. Most of the students were enrolled full-time, with a higher percentage of part-time students and employed students at the universities compared with ETHZ (S1 Table).

Twenty-two percent of the participants reported having used prescription or recreational drugs at least once for the purpose of improving cognitive performance while studying. Additionally, 12% reported the nonmedical use of prescription drugs, and 14% reported using alcohol and illegal drugs for PCE. Alcohol, methylphenidate, cannabis, and sedatives were the substances that were most commonly used for PCE (S2 Table). A total of 498 students (16.3%) reported the use of pharmacological cognitive enhancers prior to the last exam, including prescription drugs in 218 cases (7.1%) and recreational drugs (including mainly alcohol) in 334 cases (10.9%).

### Attitudes toward pharmacological cognitive enhancement

Likert scale ratings on attitudes toward PCE are listed in Table 1 for all participants and separately for users and nonusers of pharmacological cognitive enhancers, together with the test statistics. Additionally, numbers (percentages) of subjects who agreed or disagreed with a statement are shown in S3 Table.

**Table 1:** Students‘ attitudes toward pharmacological cognitive enhancement (PCE).

	Total (2,999)	PCE prior to the last exam (*223*)	PCE but not prior to the last exam (425)	Never used PCE (2,351)	Kruskal-Wallis test: H =
**Acceptability of substances used while studying (assuming rather weak efficacy)**	N = 2,933	n = 223	n = 425	n = 2,285	
Coffee	**4.2 (1.1)**	**4.3 (1.1)**	**4.3 (1.1)**	**4.1 (1.1)**	17, P<0.001
Energy drinks	**3.7 (1.3)**	**3.9 (1.3)**	**3.9 (1.3)**	**3.7 (1.3)**	18, P<0.001
Caffeine tablets	**3.1 (1.3)**	**3.6 (1.4)** *	**3.3 (1.3)** *	3.0 (1.3)	56, P<0.001
Methylphenidate or modafinil as a prescribed medication	**2.4 (1.4)**	3.0 (1.5)	**2.7 (1.5)**	**2.2 (1.4)**	89, P<0.001
Methylphenidate or modafinil without a prescription	**1.6 (0.9)**	**2.3 (1.4)** *	**1.9 (1.2)** *	**1.4 (0.9)**	89, P<0.001
Illegal stimulants (amphetamine, cocaine, or MDMA)	**1.3 (0.7)**	**1.9 (1.2)** *	**1.5 (1.0)** *	**1.3 (0.7)**	162, P<0.001
**Case example of a student using methylphenidate while studying**	N = 2,880	n = 222	n = 419	n = 2,239	
It is acceptable to use methylphenidate without a prescription	**2.0 (1.2)**	2.9 (1.6)*	**2.5 (1.4)** *	**1.9 (1.1)**	156, P<0.001
It is acceptable with a prescription even if no mental disorder has been diagnosed	**2.3 (1.3)**	2.8 (1.5)*	**2.6 (1.4)** *	**2.2 (1.2)**	73, P<0.001
It is acceptable with a prescription and with a diagnosed mental disorder	**4.0 (1.2)**	**4.1 (1.2)**	**4.1 (1.2)**	**4.0 (1.2)**	12, P<0.001
**Contexts of PCE use (assuming hypothetically strong effects & availability)**	N = 2,772	n = 209	n = 392	n = 2,171	
PCE is fair if fellow students use effective substances	**2.0 (1.2)**	2.8 (1.5)*	**2.4 (1.4)** *	**1.9 (1.1)**	115, P<0.001
PCE is fair if fellow students have poor grades	**2.1 (1.3)**	2.9 (1.5)*	**2.5 (1.4)** *	**2.0 (1.2)**	112, P<0.001
PCE is fair if fellow students have a diagnosed mental disorder	**3.8 (1.1)**	**4.1 (1.1)** *	**3.9 (1.1)** *	**3.7 (1.1)**	37, P<0.001
PCE is fair if fellow students have a mental disorder but no diagnosis	**2.6 (1.3)**	**3.2 (1.4)** *	2.9 (1.4)*	**2.5 (1.3)**	82, P<0.001
PCE is fair if surgeons have long operations or night shifts	**2.7 (1.4)**	**3.1 (1.6)** *	2.9 (1.5)*	**2.6 (1.4)**	32, P<0.001
PCE is fair if professors improve their academic teaching skills/ learning	**2.1 (1.3)**	2.8 (1.5)*	**2.5 (1.4)** *	**2.0 (1.2)**	96, P<0.001
**General attitudes toward PCE**	N = 2,601	n = 195	n = 366	n = 2,040	
PCE negatively alters the quality of the academic result	**2.8 (1.1)**	**2.7 (1.2)**	**2.8 (1.1)**	**2.9 (1.1)**	NS
PCE changes feelings and social behaviour	**3.7 (1.0)**	**3.7 (1.1)**	**3.8 (1.0)**	**3.7 (0.9)**	NS
PCE changes the personality	**3.3 (1.1)**	3.1 (1.2)	**3.3 (1.1)**	**3.4 (1.0)**	NS
Results obtained with PCE are less worthy of recognition	**3.9 (1.3)**	3.1 (1.6)*	**3.5 (1.4)** *	**4.0 (1.2)**	83, P<0.001
Someone who needs PCE poorly planned his work	**3.2 (1.3)**	**2.6 (1.4)** *	2.9 (1.3)*	**3.3 (1.3)**	77, P<0.001
PCE hinders acquisition of planning and learning skills	**3.6 (1.2)**	3.1 (1.4)*	**3.4 (1.3)** *	**3.7 (1.1)**	56, P<0.001
Mankind always used substances to enhance performance, PCE is not new	**2.5 (1.3)**	3.1 (1.4)*	2.8 (1.4)*	**2.4 (1.2)**	68, P<0.001
PCE is generally unproblematic and acceptable	**1.8 (1.0)**	**2.4 (1.3)** *	**2.1 (1.1)** *	**1.7 (0.9)**	103, P<0.001
PCE is unproblematic as long as the substances are safe	**2.3 (1.3)**	2.8 (1.4)*	**2.7 (1.3)** *	**2.2 (1.2)**	72, P<0.001
PCE is unproblematic as long as the use occurs controlled and occasionally	**2.6 (1.3)**	3.1 (1.4)*	3.0 (1.4)*	**2.4 (1.2)**	95, P<0.001
PCE is unproblematic as long as it causes no harm to others	**2.4 (1.3)**	3.0 (1.5)*	2.8 (1.4)*	**2.3 (1.2)**	87, P<0.001
**Comparison with doping in sports**	N = 2,561	n = 191	n = 357	n = 2,013	
PCE in academia is similar to doping in sports	**3.7 (1.3)**	**3.2 (1.5)** *	**3.3 (1.4)** *	**3.8 (1.3)**	59, P<0.001
In sports there are winners and loosers, PCE does not necessarily result in loosers	**2.8 (1.3)**	3.1 (1.5)*	3.0 (1.3)*	**2.7(1.2)**	36, P<0.001
PCE is acceptable in a non-competitive environment with no relative ratings	3.0 (1.4)	**3.6 (1.3)** *	**3.3 (1.4)** *	3.0 (1.3)	47, P<0.001
PCE is acceptable in a competitive environment with relative ratings	**1.7 (1.1)**	**2.4 (1.4)** *	**2.0 (1.2)** *	**1.6 (0.9)**	91, P<0.001
**Autonomy vs. Regulation**	N = 2,524	n = 189	n = 352	n = 1,983	
Everyone is free to decide whether to use drugs for PCE or not	**3.5 (1.4)**	**3.9 (1.2)** *	**3.8 (1.3)** *	**3.5 (1.4)**	31, P<0.001
I do not care if others use drugs for PCE while studying	3.0 (1.3)	**3.6 (1.4)** *	**3.3 (1.4)** *	2.9 (1.3)	66, P<0.001
The university should should regulate drug use for PCE	2.9 (1.4)	**2.5 (1.4)** *	**2.6 (1.3)** *	3.0 (1.3)	49, P<0.001
Not regulating PCE might result in unequal educational and professional perspectives	**3.5 (1.2)**	3.1 (1.4)*	3.2 (1.3)*	**3.6 (1.2)**	36, P<0.001
Non-regulated access to NE substances increases the pressure to use substances	**3.2 (1.4)**	3.0 (1.5)	3.2 (1.4)	**3.2 (1.4)**	NS
Access to drugs is regulated sufficiently, specific PCE regulations are unnecessary	**2.5 (1.2)**	2.9 (1.4)*	**2.7 (1.2)** *	**2.4 (1.1)**	32, P<0.001
**Duties of academic institutions**	N = 2,502	n = 188	n = 345	n = 1,969	
PCE use at universities may increase performance requirements	**3.4 (1.2)**	**3.5 (1.4)**	**3.3 (1.3)**	**3.4 (1.2)**	NS
We should invest more in research to develop effective substances for PCE	**2.2 (1.2)**	**2.8 (1.5)** *	**2.5 (1.3)** *	**2.1 (1.2)**	74, P<0.001
The university should collect data on prevalence and acceptance of PCE	**3.8 (1.1)**	**3.8 (1.2)**	**3.8 (1.1)**	**3.8 (1.1)**	NS
The university should inform students about PCE and associated risks	**4.1 (1.0)**	**4.2 (1.1)**	**4.1 (1.0)**	**4.1 (1.0)**	NS
I would use safe drugs for PCE even if I gained an advantage over others	**2.1 (1.3)**	**3.2 (1.5)** *	**2.6 (1.4)** *	**1.8 (1.1)**	252, P<0.001
PCE is a media-hype with no relevance to society	**2.2 (1.1)**	2.2 (1.1)	**2.2 (1.1)**	**2.2 (1.0)**	NS

Values are mean (*SD*) of Likert scale rankings from 1 = strongly disagree, 2 = rather disagree, 3 = unsure, 4 = rather agree, 5 = strongly agree; bold numbers indicate ratings > 3 = agree, or < 3 = disagree, p<0.001 (*t*-test for single means);

*significant difference (p<0.001) compared to nonusers

Attitudes toward substance use for cognitive enhancement depended on the type of substance used (Table 1). Freely available substances, such as coffee and energy drinks, were most acceptable when used while studying. Caffeine tablets were also rated as rather acceptable (Table 1). The use of methylphenidate or modafinil for PCE was not advocated, especially when used without a prescription. Illegal stimulants were the least acceptable type of drug for PCE. Attitudes toward PCE were strongly related to personal experiences with PCE. Assuming weak efficacy, drug use for PCE was rated as more acceptable by experienced users compared with nonusers (Table 1).

In the case vignette of “Thomas,” nonmedical methylphenidate use for PCE was considered unacceptable, except by students who used PCE prior to the last exam (Table 1). Moreover, 38% of students who engaged in PCE prior to the last exam and 25% of students who engaged in PCE overall agreed with using non-prescribed methylphenidate for PCE, whereas only 9% of nonusers agreed (S3 Table). Students were slightly more tolerant of methylphenidate use for PCE if it was prescribed by a physician, even without having a diagnosed mental disorder (*Z* = 13.5, *p* < 0.001). When prescribed based on a diagnosed attention deficit disorder, methylphenidate use was the most acceptable and clearly more acceptable than non-prescribed use (*Z* = 40.9, *p* < 0.001) or prescribed use without a diagnosis (*Z* = 40.5, *p* < 0.001; Table 1). Students who engaged in PCE considered the use of methylphenidate for PCE in the example of “Thomas” as more acceptable than nonusers, independent of whether it had been prescribed or not (Table 1). Multiple regression analysis revealed that agreement with PCE was more likely among students who were experienced with PCE (*F* = 246.4, *p* < 0.001) and among male students (*F* = 448.0, *p* < 0.001), whereas age, the number of semesters studied, and being employed had no influence on whether students agreed with PCE.

For the hypothetical case of legally available substances with strong effects on cognitive performance, PCE was considered acceptable only by fellow students with a diagnosis of a mental disorder (Table 1). Pharmacological cognitive enhancement by fellow students with mental disorders that were diagnosed by a physician (*Z* = 40.8, *p* < 0.001) or without a formal diagnosis (*Z* = 25.1, *p* < 0.001) were considered more acceptable compared with PCE by any fellow student, and a formal diagnosis enhanced the acceptability of PCE (Table 1). Interestingly, PCE was considered slightly less unacceptable by fellow students with bad grades (*Z* = 7.8, *p* < 0.001) compared with PCE by fellow students in general. Students rather disagreed with substance use for enhanced performance by surgeons, but disagreement with PCE by academic professors was even more pronounced (*Z* = 23.7, *p* < 0.001). In all six contexts of use that were presented in the survey, students who were experienced with PCE were more likely to agree with substance use for performance enhancement compared with nonusers (Table 1).

Respondents did not support the view that PCE negatively alters the quality of an academic result (Table 1). Nevertheless, students who had never used drugs for PCE considered results that are achieved with PCE as less worthy of recognition (Table 1). Additionally, students were concerned that PCE might change the feelings and behavior of users and potentially affect their personality. Students who never used drugs for PCE were more likely to agree with the statement that users of pharmacological cognitive enhancers did not plan their work sufficiently and that acquiring planning and learning skills was hindered by PCE. However, more than half of students who used drugs for PCE prior to their last exam disagreed that PCE is associated with poorly planned work. Neither users nor nonusers of pharmacological cognitive enhancers considered PCE unproblematic and acceptable overall. Both users and nonusers disagreed with the statement that PCE was unproblematic, as long as the substances that are used are safe. Even controlled and occasional PCE was not rated unproblematic. Finally, the view that an individual’s use of pharmacological cognitive enhancers was unproblematic as long as no negative consequences for others occurred was not supported by nonusers, whereas students with experience of PCE had no clear opinion (Table 1).

Sixty-four percent of the participants considered PCE in academia as similar to doping in sports (S3 Table). Importantly, PCE was considered unacceptable in a competitive environment but clearly more acceptable in a non-competitive environment (*Z* = 34.1, *p* < 0.001). However, students who were experienced with PCE considered drug use for PCE as more acceptable than nonusers in both environments (Table 1).

The participants agreed that everyone should be free to decide whether to use drugs for PCE or not. Nonusers indicated that they would care if other students used drugs for PCE while studying, whereas students who were experienced with PCE cared less about others’ PCE (Table 1). Only nonusers supported the statement that unregulated access to pharmacological cognitive enhancers might result in increased pressure to use drugs for PCE. The participants disagreed with the view that access to substances for PCE is sufficiently controlled by existing regulations for medications and substance use and that no specific regulations would be needed for PCE. Nonusers were concerned that a lack of regulation might result in inequality in terms of educational and professional development. However, they were unsure about whether the university should regulate drug use for PCE (Table 1).

The participants tended to believe that PCE at universities may increase performance requirements. Both users and nonusers of pharmacological cognitive enhancers similarly agreed that the university should collect data on the prevalence and acceptance of drug use for PCE among students. Furthermore, all of the students agreed that the university should inform students neutrally about PCE and potential risks associated with such behavior. Finally, the majority of nonusers (77%) indicated that they were not interested in PCE, even if safe substances were available (S3 Table).

## Discussion

The present study had two main findings. First, Swiss university students presented rather negative attitudes toward PCE, considering the use of pharmacological cognitive enhancers fair only when prescribed by a physician for the treatment of a mental disorder. Second, students who used drugs for PCE reported higher acceptability of drug use for PCE compared with nonusers, independent of the context of use.

The present large and quantitative study showed that students raised concerns about PCE, such as medical safety, fairness, and coercion, as previously established in the literature [5]. Very few students agreed with the non-prescribed use of methylphenidate or modafinil for PCE (7%). The students agreed with PCE by fellow students only when they had a mental disorder that was diagnosed by a physician (64%) but not when they had an assumed disorder (i.e., attention deficit disorder) without a formal diagnosis by a physician (26%). Students who were experienced with PCE were more likely to agree with every form of substance use for PCE compared with nonusers, confirming our primary hypothesis and the findings of previous studies [5,7].

### Medical safety and effects on personality

Many studies have weighed the benefits of PCE against its potential harms and found that nonusers were more concerned, and PCE users overestimated the beneficial cognitive-enhancing effects [5]. Our findings revealed that medical safety was not the main concern about why PCE is considered unacceptable. Nonusers agreed that PCE was problematic even if the substances were safe, even if the use was controlled and occurred only occasionally (i.e., low risk of dependence), and even if there were no negative consequences that could affect others (e.g., impaired driving). Most nonusers indicated that they were unwilling to use hypothetically safe drugs for PCE if they consequently gained an advantage over others (77%). Thus, considerations of fairness (also see below) rather than a fear of adverse effects appears to be the reason for not using PCE. A previous study among German students found that the willingness to use drugs for PCE decreased as the likelihood of expected side effects increased [3]. Similarly, a previous Swiss study found that 85% of students worried about potential side effects of drugs that are currently reported as being used for PCE [7]. Only one German study has presented contrasting findings to date, showing that the majority of surveyed students would consider using drugs for PCE if such drugs were safe [19]. Nevertheless, the present data indicate that other considerations (see below) may be as important as safety of use [3]. Thus, considering the views of the students, any regulations of drug use for PCE cannot be solely legitimized by potential health risks.

A significant proportion of the students in the present study agreed with the opinion that drug use for PCE alters users’ feelings and social behavior (61%) and even personality (46%). Consistent with this finding, half of the students in a previous Swiss study who were inexperienced with PCE and one-third of the students who were experienced with PCE indicated that they had concerns about users “not being themselves anymore” [7]. Because the use of pharmacological cognitive enhancers while studying mainly occurs during exam periods and in low quantities [6,15], substantial personality changes seem very unlikely. However, the perception and subjective belief that changes in personality can occur are real concerns [7] that might be explained by a lack of information on the main drugs that are discussed as cognitive enhancers.

### Coercion

A concern of PCE is that other people may be pressured or coerced into using drugs for PCE when taking notice of colleagues’ use. However, less than 3% of the students in our previous study considered PCE as justifiable based solely on others’ use [6]. Nonetheless, peer pressure and high demands on cognitive functioning in a competitive environment were previously associated with increased approval of PCE [20,21]. More than half of the students who participated in the present study (56%) agreed that PCE may increase performance requirements at universities. However, opinions about whether academic institutions have a duty to regulate drug use for PCE were divided (equal proportions agreed and disagreed). Combined with the findings that students were unsure about the effects of PCE on the quality of work and that a large majority of students agreed that academic institutions should inform them about PCE and associated risks, we suggest that coercion is a concern that results from a poor understanding of the effects and risks of the drugs that are currently used for PCE. This is an important finding of the present study, calling for providing students with more evidence-based information on PCE.

### Fairness

Overall, the use of hypothetically effective drugs for PCE by fellow students was considered unfair by 70% of all respondents. A previous study found that 53% of German students considered PCE by fellow students unfair [14]. As expected, students who used drugs while studying prior to the last exam (35%) and other PCE-experienced students (24%) were more likely to consider that PCE was fair compared with nonusers (11%). This finding is consistent with previous research [5,7].

Furthermore, academic doping has often been compared with sports in terms of concerns about fairness in the ethical debate about performance enhancement [10,12]. In the present study, more than half of the students (58%) agreed that PCE in academia was similar to doping in sports. In contrast, in a recent study, doping in sports was considered less ethical than academic doping [12]. The authors argued that this result indicates that individuals might have been aware of the fact that intelligence associated with academic success was less malleable than physical condition.

Moreover, fellow students’ use of hypothetically effective drugs for PCE was considered fair only when fellow students were diagnosed with a mental disorder by a physician. Using drugs for PCE while having an assumed disorder (i.e. self-diagnosed attention deficit problems) but without a physician’s diagnosis was considered much less fair. Similar to a previous study among German students [14], only a minority of students in the present study approved PCE among professors and surgeons (i.e., two professions with a high level of responsibility). Thus, students in Switzerland do not think that people with jobs that are associated with a high level of responsibility should cognitively enhance themselves [22].

Most of the participants in the present study (68%) agreed with the statement that results that are obtained with PCE are less worthy of recognition. This finding was consistent with other studies, indicating that achievements with PCE were considered less commendable than performance that is achieved without such PCE [5,14]. A study among Swiss students showed that nonusers were more concerned about not being proud of their achievements when having used drugs for PCE compared with experienced users (42% *vs*. 19%) [7]. These indirect costs of PCE because of nonusers’ concerns about the authenticity of performance while under the influence of pharmacological cognitive enhancers might decrease the well-being of users [23].

### Limitations

Because the response rate for the present survey was 10%, the study sample may not have been necessarily representative of all Swiss students. Although all students from UniBas and ETHZ were invited, only 5,000 of a total of 26,000 students who are currently enrolled at UZH who had previously agreed to be contacted for participation in various studies could be invited. Because the primary aim of the present study was to compare attitudes toward PCE between users and nonusers, the lack of representativeness of the study population for all students is not considered to be critically important. The strengths of the present study include its large sample size and the inclusion of both nonusers and two different groups of students who were experienced with PCE. The sample sizes in other studies that investigated attitudes toward PCE were often small [5] and sometimes limited to nonusers [13,15].

## Conclusions

Swiss students’ main concerns about PCE were coercion and fairness. As expected, these concerns were more prevalent among nonusers compared with students who were experienced with PCE. More balanced information on PCE should be provided to students, and the future monitoring of PCE is recommended.

## Supporting Information

S1 Questionnaire (DOCX)

S1 Table Response rate and participant characteristics. (DOC)

S2 Table Prescription and recreational drug use for pharmacological cognitive enhancement (PCE) while studying. (DOC)

S3 Table Students’ attitudes toward pharmacological cognitive enhancement (PCE) in numbers and proportions. (XLSX)
