# Supplementary material for: Swiss University Students’ Attitudes toward Pharmacological Cognitive Enhancement
Source: PLoS One. 2015 Dec 10;10(12):e0144402. doi: 10.1371/journal.pone.0144402 (PMC4675521; doi:10.1371/journal.pone.0144402)
Supplement: S1 Questionnaire — (DOCX) [file pone.0144402.s001.docx]

**S1 Questionnaire**

**Ist Hirndoping fair? Einstellungen zu Neuro-Enhancement im Studium**

Liebe Studierende der Universität Zürich, Universität Basel und ETH Zürich

Hirndoping (Neuro-Enhancement) mit Medikamenten oder psychoaktiven Substanzen, um besser Lernen zu können, kommt bei Studierenden vor. Wir wissen aber nicht wie Studierende zum Hirndoping stehen. Da wir mehr über die **Einstellung** zum Hirndoping von Studierenden wissen möchten, brauchen wir Ihre Unterstützung. Dabei spielt es keine Rolle, ob Sie im Prüfungsstress schon einmal zu „Helferchen“ etc. gegriffen haben oder nicht. Sie können auch eine Meinung zum Hirndoping haben, ohne dass sie jemals selber damit konfrontiert waren.

Unter Hirndoping verstehen wir in dieser Studie die gezielte Einnahme von Medikamenten (z.B. Ritalin®) oder psychoaktiven Substanzen (z.B. Alkohol, Cannabis, Kokain), um eine **Verbesserung der Gehirnleistung** (z.B. Aufmerksamkeit, Konzentration, Wachheit, Reduktion der Nervosität) im Kontext des Studiums zu erzielen. Kaffee und Energy Drinks, die ebenfalls konzentrationsfördernd wirken können, werden von dieser Definition ausgeschlossen.

Die Beantwortung des Fragebogens wird **ca. 15 Minuten** in Anspruch nehmen. Bitte nehmen Sie sich genügend Zeit, um alle Fragen gründlich durchzulesen und ehrlich zu beantworten. Ihre Teilnahme an dieser Studie ist freiwillig. Bitte antworten Sie auf **alle** Fragen, auch wenn diese nicht auf Sie zutreffen. Ihre Antworten sind vollständig anonym und werden anonym auf einem Sicherheitsserver gespeichert. Auch bei der Auswertung der Daten können keine Rückschlüsse auf die einzelnen Personen gezogen werden.

Die Studie wurde von der Ethikkommission beider Basel und der Ethikkommission der Philosophischen Fakultät der Universität Zürich bewilligt.

Bei Fragen können Sie sich gerne an [matthias.liechti@usb.ch](mailto:matthias.liechti@usb.ch) wenden.

Herzlichen Dank für Ihre Unterstützung und Ihre Teilnahme an dieser wichtigen Umfrage.

PD Dr. Michael Schaub, vom Schweizer Institut für Sucht- und Gesundheitsforschung Zürich & Prof. Matthias Liechti, vom Universitätsspital Basel

Indem Sie auf „weiter“ klicken bestätigen Sie, dass Sie damit einverstanden sind, dass die gemachten Angaben im Rahmen der Auswertung der Studie verwendet werden. Ihre Antworten erfolgen vollständig anonym, es sind keine Rückschlüsse auf Ihre Person möglich.

Weiter

Zuerst bitten wir Sie, einige Fragen zu Ihrem Studium und Ihrer Person zu beantworten.

(Die Überschriften dienen als Page-Title im Onlinefragebogen. Alle Fragen bis zur nächsten Überschrift werden auf *einer* Webpage untergebracht.)

### Demographische Angaben (1)

1. An welcher Hochschule studieren Sie?
   1. Universität Zürich (gehe zu 2)
   2. Universität Basel (gehe zu 4)
   3. ETH Zürich (gehe zu 3)
2. Was ist ihr Hauptfach? (falls Antwort Universität Zürich)
   1. Biologie
   2. Chemie
   3. Medizin
   4. Politikwissenschaften
   5. Psychologie
   6. Publizistik- und Kommunikationswissenschaften
   7. Rechtswissenschaften
   8. Sozialwissenschaften
   9. Wirtschaftswissenschaften
   10. Veterinärmedizin
   11. Anderes [Eingabe]
3. Was ist ihr Hauptfach? (falls Antwort ETH)
   1. Architektur
   2. Bewegungswissenschaften und Sport
   3. Biologie
   4. Chemie
   5. Informatik
   6. Maschineningenieurwissenschaften
   7. Mathematik
   8. Pharmazeutische Wissenschaften
   9. Physik
   10. Umweltnaturwissenschaften
   11. Anderes [Eingabe]
4. Was ist ihr Hauptfach? (falls Antwort Universität Basel)
   1. Biologie
   2. Chemie
   3. Medizin
   4. Pädagogik
   5. Pharmazeutische Wissenschaften
   6. Politikwissenschaften
   7. Psychologie
   8. Publizistik- und Kommunikationswissenschaften
   9. Rechtswissenschaften
   10. Sprachwissenschaften
   11. Wirtschaftswissenschaften
   12. Veterinärmedizin
   13. Anderes [Eingabe]

## Demographische Angaben (2)

1. In welchem Semester sind Sie?
2. Bitte geben Sie ihre Antwort hier ein: [Eingabe]
3. Wie alt sind Sie?
   1. [Eingabe] Jahre

1. Geschlecht?
   1. weiblich
   2. männlich
2. Studieren Sie Vollzeit oder Teilzeit?
   1. Vollzeit
   2. Teilzeit
3. Arbeiten Sie neben dem Studium?
   1. Ja
   2. Anzahl Stellenprozent [Eingabe]
   3. Nein

## Hirndoping – Bekanntheit und Verbreitung

1. Haben Sie schon davon gehört, dass man verschreibungspflichtige Medikamente oder psychoaktive Substanzen (Drogen) zur Verbesserung der Gehirnleistung (Hirndoping) konsumieren kann.

a. Ja

b. Nein

1. Wie viele Personen kennen Sie, die im Studium verschreibungspflichtige Medikamente ohne medizinische Notwendigkeit zur Verbesserung der Gehirnleistung (Hirndoping) im Studium konsumieren oder konsumiert haben? (Zum Beispiel Ritalin®, um effizienter oder länger lernen zu können.)
   1. 1-2 Personen
   2. 3-5 Personen
   3. 6-8 Personen
   4. mehr als 8 Personen
   5. niemanden
2. Wie viele Personen kennen Sie, die im Studium psychoaktive Substanzen (z.B. Alkohol, Cannabis, Kokain) konsumieren oder konsumiert haben, um eine direkte oder indirekte Verbesserung der Gehirnleistung (z.B. Aufmerksamkeit, Konzentration, Wachheit, Reduktion der Nervosität) im Kontext des Studiums zu erzielen.
   1. 1-2 Personen
   2. 3-5 Personen
   3. 6-8 Personen
   4. mehr als 8 Personen
   5. niemanden

## Verschreibungspflichtige Medikamente als Hirndoping

1. Haben Sie jemals im Leben eines oder mehrere der unten aufgeführten Medikamente **zur Verbesserung der Gehirnleistung im Studium oder Gymnasium** eingenommen, ohne dass eine klare medizinische Indikation vorlag (ohne eine spezifische Erkrankung)? Zum Beispiel, um in Stresssituationen in der Prüfungszeit bessere Leistungen zu erbringen oder um effizienter oder länger lernen zu können oder um vor der Prüfung besser schlafen und entspannen zu können.
   1. **Methylphenidat** (Ritalin®, Concerta®, Medikinet®, Focalin®, Strattera®)
      1. Ja
      2. Nein

Wie oft in den letzten 30 Tagen vor der letzten grossen Prüfung bzw. dem letzten grossen Leistungsnachweis?

An 20 oder mehr Tagen, an 10-19 Tagen, An 4-9 Tagen, An 1-3 Tagen, Gar nicht

Wurden ihre Erwartungen an die Wirkung der Substanz erfüllt?

i. Ja

ii. Nein

iii. Weiss nicht

Erwägen Sie eine wiederholte Einnahme in einer ähnlichen Situation?

i. Ja

ii. Nein

- 1. **Modafinil** (Modasomil®)
     1. Ja
     2. Nein

Wie oft in den letzten 30 Tagen vor der letzten grossen Prüfung bzw. dem letzten grossen Leistungsnachweis?

An 20 oder mehr Tagen, an 10-19 Tagen, An 4-9 Tagen, An 1-3 Tagen, Gar nicht

Wurden ihre Erwartungen an die Wirkung der Substanz erfüllt?

i. Ja

ii. Nein

iii. Weiss nicht

Erwägen Sie eine wiederholte Einnahme in einer ähnlichen Situation?

i. Ja

ii. Nein

- 1. **Aktivierende Antidepressiva** (Edronax®, Wellbutrin®, Efexor®, Cymbalta®, Fluctine®/Fluoxetin, Seropram®/Citalopram, Cipralex®, Zoloft®/Sertralin)
     1. Ja
     2. Nein

Wie oft in den letzten 30 Tagen vor der letzten grossen Prüfung bzw. dem letzten grossen Leistungsnachweis?

An 20 oder mehr Tagen, an 10-19 Tagen, An 4-9 Tagen, An 1-3 Tagen, Gar nicht

Wurden ihre Erwartungen an die Wirkung der Substanz erfüllt?

i. Ja

ii. Nein

iii. Weiss nicht

Erwägen Sie eine wiederholte Einnahme in einer ähnlichen Situation?

i. Ja

ii. Nein

- 1. **Antidementiva** (z.B. Aricept®, Axura®, Reminyl®, Exelon®)
     1. Ja
     2. Nein

Wie oft in den letzten 30 Tagen vor der letzten grossen Prüfung bzw. dem letzten grossen Leistungsnachweis?

An 20 oder mehr Tagen, an 10-19 Tagen, An 4-9 Tagen, An 1-3 Tagen, Gar nicht

Wurden ihre Erwartungen an die Wirkung der Substanz erfüllt?

i. Ja

ii. Nein

iii. Weiss nicht

Erwägen Sie eine wiederholte Einnahme in einer ähnlichen Situation?

i. Ja

ii. Nein

- 1. **Beruhigungs- und Schlafmittel, Hypnotica** (z.B. Remeron®, Sonata®, Stilnox®, Imovane®, Dalmadorm®, Noctamid®, Ergocalm Tabs®, Remestan®, Halcion®, Dolestan®, Chloraldurat 500®, Temesta®, Valium®, Xanax®)
     1. Ja
     2. Nein

Wie oft in den letzten 30 Tagen vor der letzten grossen Prüfung bzw. dem letzten grossen Leistungsnachweis?

An 20 oder mehr Tagen, an 10-19 Tagen, An 4-9 Tagen, An 1-3 Tagen, Gar nicht

Wurden ihre Erwartungen an die Wirkung der Substanz erfüllt?

i. Ja

ii. Nein

iii. Weiss nicht

Erwägen Sie eine wiederholte Einnahme in einer ähnlichen Situation?

i. Ja

ii. Nein

- 1. **Betablocker** (z.B. Beloc ZOK®, MetoZerok®, Inderal®)
     1. Ja
     2. Nein

Wie oft in den letzten 30 Tagen vor der letzten grossen Prüfung bzw. dem letzten grossen Leistungsnachweis?

An 20 oder mehr Tagen, an 10-19 Tagen, An 4-9 Tagen, An 1-3 Tagen, Gar nicht

Wurden ihre Erwartungen an die Wirkung der Substanz erfüllt?

i. Ja

ii. Nein

iii. Weiss nicht

Erwägen Sie eine wiederholte Einnahme in einer ähnlichen Situation?

i. Ja

ii. Nein

## Psychoaktive Substanzen als Hirndoping

1. Haben Sie jemals im Leben eine oder mehrere der unten aufgeführten psychoaktiven Substanzen eingenommen mit dem **Ziel der Verbesserung der Gehirnleistung im Studium oder Gymnasium**? Zum Beispiel, um effizienter oder länger lernen zu können oder um während der Prüfung wacher zu sein oder um in der Prüfungszeit besser entspannen oder schlafen zu können. Freizeitgebrauch z.B. im Ausgang oder an Partys sollte nicht eingerechnet werden.
   1. **Alkohol**
      1. Ja
      2. Nein

Wie oft in den letzten 30 Tagen vor der letzten grossen Prüfung bzw. dem letzten grossen Leistungsnachweis?

An 20 oder mehr Tagen, an 10-19 Tagen, An 4-9 Tagen, An 1-3 Tagen, Gar nicht

Wurden ihre Erwartungen an die Wirkung der Substanz erfüllt?

i. Ja

ii. Nein

iii. Weiss nicht

Erwägen Sie eine wiederholte Einnahme in einer ähnlichen Situation?

i. Ja

ii. Nein

- 1. **Hanfprodukte:** Gras, Hasch, Haschöl
     1. Ja
     2. Nein

Wie oft in den letzten 30 Tagen vor der letzten grossen Prüfung bzw. dem letzten grossen Leistungsnachweis?

An 20 oder mehr Tagen, an 10-19 Tagen, An 4-9 Tagen, An 1-3 Tagen, Gar nicht

Wurden ihre Erwartungen an die Wirkung der Substanz erfüllt?

i. Ja

ii. Nein

iii. Weiss nicht

Erwägen Sie eine wiederholte Einnahme in einer ähnlichen Situation?

i. Ja

ii. Nein

- 1. **Kokain**
     1. Ja
     2. Nein

Wie oft in den letzten 30 Tagen vor der letzten grossen Prüfung bzw. dem letzten grossen Leistungsnachweis?

An 20 oder mehr Tagen, an 10-19 Tagen, An 4-9 Tagen, An 1-3 Tagen, Gar nicht

Wurden ihre Erwartungen an die Wirkung der Substanz erfüllt?

i. Ja

ii. Nein

iii. Weiss nicht

Erwägen Sie eine wiederholte Einnahme in einer ähnlichen Situation?

i. Ja

ii. Nein

- 1. **Amphetamine (Speed)**
     1. Ja
     2. Nein

Wie oft in den letzten 30 Tagen vor der letzten grossen Prüfung bzw. dem letzten grossen Leistungsnachweis?

An 20 oder mehr Tagen, an 10-19 Tagen, An 4-9 Tagen, An 1-3 Tagen, Gar nicht

Wurden ihre Erwartungen an die Wirkung der Substanz erfüllt?

i. Ja

ii. Nein

iii. Weiss nicht

Erwägen Sie eine wiederholte Einnahme in einer ähnlichen Situation?

i. Ja

ii. Nein

- 1. **Ecstasy** (MDMA)
     1. Ja
     2. Nein

Wie oft in den letzten 30 Tagen vor der letzten grossen Prüfung bzw. dem letzten grossen Leistungsnachweis?

An 20 oder mehr Tagen, an 10-19 Tagen, An 4-9 Tagen, An 1-3 Tagen, Gar nicht

Wurden ihre Erwartungen an die Wirkung der Substanz erfüllt?

i. Ja

ii. Nein

iii. Weiss nicht

Erwägen Sie eine wiederholte Einnahme in einer ähnlichen Situation?

i. Ja

ii. Nein

## Einstellung zu Neuro-Enhancement (schwache Wirksamkeit vermutet)

Wir bitten Sie nun, Fragen zu Ihrer Einstellung zum Neuro-Enhancement zu beantworten.

Für die Fragen auf dieser Seite gehen Sie bitte davon aus, dass die genannten Substanzen in der Regel eine **eher schwache Wirkung** auf die kognitive Leistungsfähigkeit haben. (Dies entspricht in Wahrheit auch der aktuellen wissenschaftlichen Befundlage)

1. Beurteilen Sie für jede der nachfolgend genannten bekannten Substanzen/Situationen, **wie sehr Sie die Verwendung zur Verbesserung der Gehirnleistung im Studium befürworten.**

*(Auf einer Skala von 1-5: 1 = ich stimme überhaupt nicht zu, 5 = ich stimme vollkommen zu)*.

- 1. Verschreibungspflichtiges Methylphenidat (Ritalin^®^, Concerta^®,^ Medikinet®, Focalin®, Strattera®) oder Modafinil (Modasomil®)
     1. als Medikament mit ärztlichem Rezept
        1. Ich stimme überhaupt nicht zu
        2. Ich stimme eher nicht zu
        3. Ich bin unsicher
        4. Ich stimme eher zu
        5. Ich stimme vollkommen zu
     2. Verwendung ohne ärztliches Rezept
        1. Ich stimme überhaupt nicht zu
        2. Ich stimme eher nicht zu
        3. Ich bin unsicher
        4. Ich stimme eher zu
        5. Ich stimme vollkommen zu
  2. Illegale Stimulanzien (Amphetamin, Kokain, Ecstasy)
     - 1. Ich stimme überhaupt nicht zu
       2. Ich stimme eher nicht zu
       3. Ich bin unsicher
       4. Ich stimme eher zu
       5. Ich stimme vollkommen zu
  3. Kaffee
     - 1. Ich stimme überhaupt nicht zu
       2. Ich stimme eher nicht zu
       3. Ich bin unsicher
       4. Ich stimme eher zu
       5. Ich stimme vollkommen zu
  4. Koffeintabletten
     - 1. Ich stimme überhaupt nicht zu
       2. Ich stimme eher nicht zu
       3. Ich bin unsicher
       4. Ich stimme eher zu
       5. Ich stimme vollkommen zu
  5. Energy Drinks
     - 1. Ich stimme überhaupt nicht zu
       2. Ich stimme eher nicht zu
       3. Ich bin unsicher
       4. Ich stimme eher zu
       5. Ich stimme vollkommen zu

## Einstellung zu Neuro-Enhancement (Fallbeispiel)

Nun folgt ein Fallbeispiel. Bitte lesen Sie dieses genau durch und sagen Sie anschliessend, wie sehr Sie den nachfolgenden Aussagen zustimmen.

*(Auf einer Skala von 1-5: 1 = ich stimme überhaupt nicht zu, 5 = ich stimme vollkommen zu)*.

1. Thomas lernt für das Schlussexamen. Er hat Mühe, sich zu konzentrieren. Er beklagt sich bei einem Kollegen, dass er sich unter Druck fühle und Schwierigkeiten mit dem Lernen habe. Sein Freund meint, etwas Ritalin könnte helfen und bietet ihm welches an. Thomas nimmt das Ritalin und empfindet es einfacher, sich damit länger zu konzentrieren. Thomas hat das Gefühl, effizienter zu lernen und hat weniger Angst vor dem Examen. Thomas besteht das Examen mit etwas besseren Noten als bisher.
   1. Es ist akzeptabel, dass Thomas in dieser Situation Ritalin verwendet.
      1. Ich stimme überhaupt nicht zu
      2. Ich stimme eher nicht zu
      3. Ich bin unsicher
      4. Ich stimme eher zu
      5. Ich stimme vollkommen zu
   2. Es ist akzeptabel, dass Thomas in dieser Situation Ritalin verwendet, falls er dieses **mit ärztlicher Verschreibung** erhält, auch wenn **keine Krankheit diagnostiziert** wurde.
      1. Ich stimme überhaupt nicht zu
      2. Ich stimme eher nicht zu
      3. Ich bin unsicher
      4. Ich stimme eher zu
      5. Ich stimme vollkommen zu
   3. Es ist akzeptabel, dass Thomas in dieser Situation Ritalin verwendet falls er dieses **mit ärztlicher Verschreibung** im Zusammenhang mit der **Diagnose einer Aufmerksamkeitsstörung** erhält.
      1. Ich stimme überhaupt nicht zu
      2. Ich stimme eher nicht zu
      3. Ich bin unsicher
      4. Ich stimme eher zu
      5. Ich stimme vollkommen zu

## Einstellung zu Neuro-Enhancement (Starke Wirksamkeit vermutet)

Für die Fragen auf dieser Seite gehen Sie bitte davon aus, dass die genannten Substanzen in der Regel **eine starke Wirkung auf die kognitive Leistungsfähigkeit** haben (Dies entspricht in Wahrheit nicht der aktuellen wissenschaftlichen Befundlage) und **für alle legal zur Verfügung stehen**.

1. Fänden Sie es unter der oben geschilderten Ausgangslage fair, wenn:
2. Mitstudierende wirksame Substanzen einnehmen, um ihre kognitive Leistung im Studium zu verbessern?
   1. Ich stimme überhaupt nicht zu
   2. Ich stimme eher nicht zu
   3. Ich bin unsicher
   4. Ich stimme eher zu
   5. Ich stimme vollkommen zu
3. Mitstudierende mit schlechten Noten wirksame Substanzen einnehmen, um ihre kognitive Leistung im Studium zu verbessern?
   - 1. Ich stimme überhaupt nicht zu
     2. Ich stimme eher nicht zu
     3. Ich bin unsicher
     4. Ich stimme eher zu
     5. Ich stimme vollkommen zu
4. Mitstudierende ***mit*** einer ärztlichen Diagnose einer psychischen Störung (z.B. der Aufmerksamkeit) wirksame Substanzen einnehmen, um ihre kognitive Leistung zu verbessern?
   - 1. Ich stimme überhaupt nicht zu
     2. Ich stimme eher nicht zu
     3. Ich bin unsicher
     4. Ich stimme eher zu
     5. Ich stimme vollkommen zu
5. Mitstudierende mit einer psychischen Störung (z.B. der Aufmerksamkeit), aber ***ohne*** eine ärztliche Diagnose, wirksame Substanzen einnehmen, um ihre kognitive Leistung zu verbessern?
   - 1. Ich stimme überhaupt nicht zu
     2. Ich stimme eher nicht zu
     3. Ich bin unsicher
     4. Ich stimme eher zu
     5. Ich stimme vollkommen zu
6. Chirurgen wirksame Substanzen einnehmen, um ihre Arbeitsleistung während langen Operationen oder während der Nacht zu verbessern?
   - 1. Ich stimme überhaupt nicht zu
     2. Ich stimme eher nicht zu
     3. Ich bin unsicher
     4. Ich stimme eher zu
     5. Ich stimme vollkommen zu
7. Professoren und Dozenten Substanzen einnehmen, um ihre akademische Lehr-/Lernleistung zu verbessern?
   - 1. Ich stimme überhaupt nicht zu
     2. Ich stimme eher nicht zu
     3. Ich bin unsicher
     4. Ich stimme eher zu
     5. Ich stimme vollkommen zu

## Einstellung zu Neuro-Enhancement (Grundsätzliches 1)

Wie stehen Sie zu den folgenden Aussagen?

*(Auf einer Skala von 1-5: 1 = ich stimme überhaupt nicht zu, 5 = ich stimme vollkommen zu)*.

1. Neuro-Enhancement verändert die Qualität des akademischen Resultates in negativer Weise (z.B. weniger originell/phantasievoll).
   1. Ich stimme überhaupt nicht zu
   2. Ich stimme eher nicht zu
   3. Ich bin unsicher
   4. Ich stimme eher zu
   5. Ich stimme vollkommen zu
2. Substanzen zur Steigerung der geistigen Leistung verändern die Gefühle und das soziale Verhalten einer Person.
3. Ich stimme überhaupt nicht zu
4. Ich stimme eher nicht zu
5. Ich bin unsicher
6. Ich stimme eher zu
7. Ich stimme vollkommen zu
8. Substanzen zur Steigerung der geistigen Leistung verändern die „Persönlichkeit“ des betreffenden Menschen.
9. Ich stimme überhaupt nicht zu
10. Ich stimme eher nicht zu
11. Ich bin unsicher
12. Ich stimme eher zu
13. Ich stimme vollkommen zu
14. Leistungen, die mit Hilfe von Substanzen zur geistigen Leistungssteigerung erbracht werden, sind weniger anerkennungswürdig als vergleichbare Leistungen, die ohne diese Substanzen erbracht wurden.
15. Ich stimme überhaupt nicht zu
16. Ich stimme eher nicht zu
17. Ich bin unsicher
18. Ich stimme eher zu
19. Ich stimme vollkommen zu
20. Wer Substanzen braucht, um länger lernen zu können, hat seine Arbeit schlecht geplant.
21. Ich stimme überhaupt nicht zu
22. Ich stimme eher nicht zu
23. Ich bin unsicher
24. Ich stimme eher zu
25. Ich stimme vollkommen zu
26. Die Verwendung von Substanzen, um besser lernen zu können, verhindert die Aneignung von Planungs- und Lernstrategien, welche nachhaltiger sind.
27. Ich stimme überhaupt nicht zu
28. Ich stimme eher nicht zu
29. Ich bin unsicher
30. Ich stimme eher zu

Ich stimme vollkommen zu

1. Die Menschheit hat seit jeher Substanzen zur Leistungssteigerung verwendet (Kaffee, Coca, Betel, Tabak). Die Verwendung neuerer Substanzen wie Medikamente (z. B. Ritalin®) ist einfach die neuste Form dieses Phänomens.
2. Ich stimme überhaupt nicht zu
3. Ich stimme eher nicht zu
4. Ich bin unsicher
5. Ich stimme eher zu
6. Ich stimme vollkommen zu

## Einstellung zu Neuro-Enhancement (Grundsätzliches 2)

1. Neuro-Enhancement ist:
2. generell kein Problem und akzeptabel.
3. Ich stimme überhaupt nicht zu
4. Ich stimme eher nicht zu
5. Ich bin unsicher
6. Ich stimme eher zu
7. Ich stimme vollkommen zu
8. kein Problem, sofern die Substanzen sicher sind.
9. Ich stimme überhaupt nicht zu
10. Ich stimme eher nicht zu
11. Ich bin unsicher
12. Ich stimme eher zu
13. Ich stimme vollkommen zu
14. kein Problem, sofern die Substanzen kontrolliert und nur gelegentlich eingenommen werden.
15. Ich stimme überhaupt nicht zu
16. Ich stimme eher nicht zu
17. Ich bin unsicher
18. Ich stimme eher zu
19. Ich stimme vollkommen zu
20. kein Problem sofern es wegen allfälligen Nebenwirkungen keine negativen Folgen für andere hat (z.B. beeinträchtigte Fahrtüchtigkeit)
21. Ich stimme überhaupt nicht zu
22. Ich stimme eher nicht zu
23. Ich bin unsicher
24. Ich stimme eher zu
25. Ich stimme vollkommen zu

## Einstellung zu Neuro-Enhancement (Vergleich mit Doping im Sport)

1. Ist Neuro-Enhancement in der Akademie wie Doping im Sport?
2. Neuro-Enhancement im akademischen Umfeld ist das Gleiche wie Doping im Sport.
3. Ich stimme überhaupt nicht zu
4. Ich stimme eher nicht zu
5. Ich bin unsicher
6. Ich stimme eher zu
7. Ich stimme vollkommen zu
8. Im Leistungssport gibt es Gewinner und Verlierer. Im akademischen Umfeld führt ein Gewinn durch Neuro-Enhancement (Studienabschluss, Note, Job) nicht unbedingt dazu, dass Andere verlieren.
9. Ich stimme überhaupt nicht zu
10. Ich stimme eher nicht zu
11. Ich bin unsicher
12. Ich stimme eher zu
13. Ich stimme vollkommen zu
14. Neuro-Enhancement bei anderen Personen ist für mich akzeptabel
15. in einem **nicht-kompetitiven** Umfeld, in welchem meine Leistung nicht relativ zu jener anderer Studierenden bewertet wird.
16. Ich stimme überhaupt nicht zu
17. Ich stimme eher nicht zu
18. Ich bin unsicher
19. Ich stimme eher zu
20. Ich stimme vollkommen zu
21. in einem **kompetitiven** Umfeld, in welchem meine Leistung relativ zu jener anderer Studierenden bewertet wird.
22. Ich stimme überhaupt nicht zu
23. Ich stimme eher nicht zu
24. Ich bin unsicher
25. Ich stimme eher zu
26. Ich stimme vollkommen zu

## Einstellung zu Neuro-Enhancement (Autonomie)

2. Jeder ist autonom und frei selbst zu entscheiden, ob er Substanzen zur Verbesserung seiner kognitiven Leistung einsetzen möchte oder nicht.
3. Ich stimme überhaupt nicht zu
4. Ich stimme eher nicht zu
5. Ich bin unsicher
6. Ich stimme eher zu
7. Ich stimme vollkommen zu
8. Mir ist es egal, wenn andere Personen Substanzen nehmen, um zu lernen.
9. Ich stimme überhaupt nicht zu
10. Ich stimme eher nicht zu
11. Ich bin unsicher
12. Ich stimme eher zu
13. Ich stimme vollkommen zu
14. Die Universität sollte Richtlinien zur Verwendung von Substanzen zur Verbesserung der kognitiven Leistung bei Studierenden erarbeiten.
15. Ich stimme überhaupt nicht zu
16. Ich stimme eher nicht zu
17. Ich bin unsicher
18. Ich stimme eher zu
19. Ich stimme vollkommen zu
20. Ein nicht regulierter Zugang zu Substanzen zur geistigen Leistungssteigerung kann zu ungleichen Chancen hinsichtlich Ausbildung und späterer Berufstätigkeit führen.
21. Ich stimme überhaupt nicht zu
22. Ich stimme eher nicht zu
23. Ich bin unsicher
24. Ich stimme eher zu
25. Ich stimme vollkommen zu
26. Ein nicht regulierter Zugang zu Substanzen zur geistigen Leistungssteigerung erhöht den Druck, diese Substanzen einzunehmen.
27. Ich stimme überhaupt nicht zu
28. Ich stimme eher nicht zu
29. Ich bin unsicher
30. Ich stimme eher zu
31. Ich stimme vollkommen zu
32. Der Zugang zu Medikamenten und anderen Substanzen ist bereits ausreichend geregelt und spezielle Regulierungen zu Neuro-Enhancement sind daher unnötig.
33. Ich stimme überhaupt nicht zu
34. Ich stimme eher nicht zu
35. Ich bin unsicher
36. Ich stimme eher zu
37. Ich stimme vollkommen zu

## Einstellung zu Neuro-Enhancement (Universitäten)

1. Die Verwendung von Neuro-Enhancement an Universitäten könnte die Leistungsanforderungen erhöhen.
2. Ich stimme überhaupt nicht zu
3. Ich stimme eher nicht zu
4. Ich bin unsicher
5. Ich stimme eher zu
6. Ich stimme vollkommen zu
7. Es sollte mehr in die Forschung zur Entwicklung wirksamer Substanzen zur Steigerung der geistigen Leistungsfähigkeit investiert werden.
8. Ich stimme überhaupt nicht zu
9. Ich stimme eher nicht zu
10. Ich bin unsicher
11. Ich stimme eher zu
12. Ich stimme vollkommen zu
13. Die Universität/ETH soll bezüglich Neuro-Enhancement:
14. Daten zur Bedeutung sammeln (Häufigkeit/Akzeptanz unter Studierenden).
15. Ich stimme überhaupt nicht zu
16. Ich stimme eher nicht zu
17. Ich bin unsicher
18. Ich stimme eher zu
19. Ich stimme vollkommen zu
20. Die Studierenden über dieses Thema und mögliche Probleme neutral informieren.
21. Ich stimme überhaupt nicht zu
22. Ich stimme eher nicht zu
23. Ich bin unsicher
24. Ich stimme eher zu
25. Ich stimme vollkommen zu

## Einstellung zu Neuro-Enhancement (Abschluss)

1. Ich würde sichere und nebenwirkungsarme Substanzen zur Verbesserung der kognitiven Leistung verwenden, auch wenn ich mich dadurch gegenüber anderen bevorteile.
2. Ich stimme überhaupt nicht zu
3. Ich stimme eher nicht zu
4. Ich bin unsicher
5. Ich stimme eher zu
6. Ich stimme vollkommen zu
7. Neuro-Enhancement ist ein Medienhype ohne wirkliche gesellschaftliche Relevanz.
8. Ich stimme überhaupt nicht zu
9. Ich stimme eher nicht zu
10. Ich bin unsicher
11. Ich stimme eher zu
12. Ich stimme vollkommen zu

## Ende der Umfrage

1. Sie sind am Ende des Fragebogens angelangt. Wir bitten Sie jetzt noch anzugeben, wie ehrlich und seriös Sie auf die Fragen geantwortet haben.
2. Unehrlich/unseriös
3. Manchmal unehrlich/unseriös
4. Manchmal ehrlich/seriös
5. Ehrlich/seriös

**Vielen Dank für Ihre Teilnahme!**
