# Supplementary material for: Swiss University Students’ Attitudes toward Pharmacological Cognitive Enhancement
Source: PLoS One. 2015 Dec 10;10(12):e0144402. doi: 10.1371/journal.pone.0144402 (PMC4675521; doi:10.1371/journal.pone.0144402)
Supplement: S1 Table — (DOC) [file pone.0144402.s002.doc]

| **Table S1.** Response rate and participant characteristics | | | | |  |  |
| --- | --- | --- | --- | --- | --- | --- |
|  | **Total (*N*=3,056)** | **ETHZ (*n*=1,632)** | **UniBas (*n*=1,067)** | **UZH (*n*=357)** | | |
| **Response rate** | 10.4%  (3,056 of 29,282) | 12.5%  (1,632 of 13,093) | 9.5%  (1,067 of 11,189) | 7.1%  (357 of 5,000) | | |
| **Sex** |  |  |  |  | | |
| Male | 51.5% (1,574) | 60.4% (985) | 34.8 % (371) | 35.3% (126) | | |
| Female | 48.5% (1,482) | 39.6% (647) | 65.2% (696) | 64.7% (231) | | |
| **Age** (*years)* | 23.3 (4.3) | 22.4 (2.9) | 24.2 (5.1) | 24.7 (6.0) | | |
| **N° semesters** | 5.8 (3.3) | 5.3 (2.9) | 6.5 (3.5) | 6.6 (3.9) | | |
| **Study workload** | |  |  |  | | |
| Full-time | 91.6% (2,798) | 97.1% (1,584) | 86.9% (927) | 80.4% (287) | | |
| Part-time | 8.4% (258) | 2.9% (48) | 13.1% (140) | 19.6% (70) | | |
| **Employment while studying** | |  |  |  | | |
| Yes | 45.2% (1,382) | 32.1% (524) | 59.9% (639) | 61.3% (219) | | |
| No | 54.8% (1,674) | 67.9% (1,108) | 40.1% (428) | 38.7% (138) | | |
| Data are % (number of students) or mean (*SD*). ETHZ: Swiss Federal Institute of Technology Zurich; UniBas: University of Basel; UZH: University of Zurich | | | | | |  |
