# Supplementary material for: Swiss University Students’ Attitudes toward Pharmacological Cognitive Enhancement
Source: PLoS One. 2015 Dec 10;10(12):e0144402. doi: 10.1371/journal.pone.0144402 (PMC4675521; doi:10.1371/journal.pone.0144402)
Supplement: S2 Table — (DOC) [file pone.0144402.s003.doc]

| **Table S2.** Prescription and recreational drug use for pharmacological cognitive enhancement (PCE) while studying (*N*=3,056) | | | | |
| --- | --- | --- | --- | --- |
|  | **Ever used** | **Last month prior to exam** | **Daily use prior to exam** | **Expectations fulfilled^1^** |
| **Prescription drugs used for PCE** | | | | |
| Methylphenidate | 6.2% (188) | 3.4% (103) | 0.7% (22) | 59.6% (112) |
| Sedatives | 4.9% (149) | 3.2% (97) | 0.3% (10) | 77.2% (115) |
| Beta-blockers | 1.9% (57) | 1.2% (35) | 0.1% (4) | 70.2% (40) |
| Antidepressants | 0.8% (24) | 0.6% (18) | 0.4% (13) | 62.5% (15) |
| Modafinil | 0.6% (19) | 0.4% (14) | 0.1% (4) | 73.7% (14) |
| Anti-dementia drugs | 0.3% (8) | 0.3% (8) | 0.1% (4) | 75.0% (6) |
|  |  |  |  |  |
| **Recreational drugs used for PCE** | | | | |
| Alcohol | 10.0% (303) | 7.2% (241) | 0.5% (14) | 63.4% (192) |
| Cannabis | 5.0% (152) | 4.0% (122) | 0.9% (26) | 61.8% (94) |
| Illegal amphetamines | 1.5% (46) | 1.0% (31) | 0.2% (6) | 65.2% (30) |
| Cocaine | 0.5% (14) | 0.3% (9) | 0.1% (2) | 64.3% (9) |
| MDMA (ecstasy) | 0.3% (9) | 0.2% (4) | 0.1% (2) | 77.8% (7) |
| Data are % (number of students)  ^1^Percent of students who used a certain substance at least once for PCE. | | | | |
